# What Doesn’t Kill You Makes You Stronger: Psychological Trauma and Its Relationship to Enhanced Memory Control

**DOI:** 10.1037/xge0000461

**Published:** 2018-07-19

**Authors:** Justin C. Hulbert, Michael C. Anderson

**Affiliations:** 1 Psychology Program, Bard College; 2 MRC Cognition and Brain Sciences Unit, University of Cambridge

**Keywords:** forgetting, inhibitory control, memory suppression, resilience, trauma

## Abstract

Control processes engaged in halting the automatic retrieval of unwanted memories have been shown to reduce the later recallability of the targets of suppression. Like other cognitive skills that benefit from practice, we hypothesized that memory control is similarly experience dependent, such that individuals with greater real-life experience at stopping retrieval would exhibit better inhibitory control over unwanted memories. Across two experiments, we found that college students reporting a greater history of trauma exhibited more suppression-induced forgetting of both negative and neutral memories than did those in a matched group who had reported experiencing little to no trauma. The association was especially evident on a test of suppression-induced forgetting involving independent retrieval cues that are designed to better isolate the effects of inhibitory control on memory. Participants reporting more trauma demonstrated greater generalized forgetting of suppressed material. These findings raise the possibility that, given proper training, individuals can learn to better manage intrusive experiences, and are broadly consistent with the view that moderate adversity can foster resilience later in life.

After traumatic experiences, survivors often experience intrusive memories that undermine their peace of mind. Innocuous stimuli have the power to evoke unwelcome memories. The jump of a child on a sidewalk may remind a veteran of a roadside attack in Afghanistan, or a dog barking may trigger memories of the night a person got news of a loved-one’s death. Intrusive memories, however, decline over time for most people (Dougall, Craig, & Baum, 1999). This observation raises a fundamental question about the nature of this remission: Why do intrusions decline? Does this remission reflect a passive forgetting that happens to all memories? Or might people’s early efforts to cope with intrusions help enhance mental functioning to handle new challenges? Put simply, is it that “Time heals all wounds” or “What doesn’t kill you, makes you stronger?”

In this article, we consider whether the remission of intrusive memories after trauma might derive, in part, from the strengthened ability to suppress retrieval through inhibitory control. Three general observations support our hypothesis. First, after a trauma, people often report suppressing unwelcome remindings so that they can regain focus (Bomyea & Lang, 2016; Ehlers, Mayou, & Bryant, 1998). If so, repeatedly engaging this control process may have far-reaching consequences (e.g., Hulbert, Henson, & Anderson, 2016). It could constitute a natural and strongly motivated case of cognitive training, facilitating the development of effective habits of control (cf. Hertel, 2004). Recovering from intrusive memories, therefore, could partially reflect improved memory inhibition, possibly explaining why those suffering from enduring posttraumatic stress disorder (PTSD) exhibit worse memory control in laboratory studies (e.g., Catarino, Küpper, Werner-Seidler, Dalgleish, & Anderson, 2015). Second, evidence for experience-dependent cortical plasticity indicates that practicing cognitive tasks can cause lasting structural changes (see, e.g., Draganski, Kherif, & Lutti, 2014; May, 2011, for reviews). Of particular relevance, Lyoo et al. (2011) found that survivors of a tragic arson event exhibited a selective increase of cortical thickness in lateral prefrontal regions a year after the trauma. Cortical thickness of the affected regions, which were broadly consistent with those engaged in retrieval suppression (Anderson & Hanslmayr, 2014), predicted recovery from PTSD symptoms, such as memory intrusions. Third, and critically, the notion that trauma, at least in moderate amounts, may have enduring benefits receives strong support from longitudinal studies that have linked cumulative lifetime adversity to improved resilience: People exposed to some adversity exhibit better mental health and resilience to adversity later in life, relative to individuals exposed to either a high amount of adversity or to no adversity (e.g., Seery, 2011; Seery, Holman, & Silver, 2010). The counterintuitive observation that adversity can be beneficial lends credence to the possibility that trauma history fosters experience-dependent changes in cognitive skills that could support resilience, although the nature of those skills remains unclear.

Here we consider whether one skill that might benefit from adversity is the ability to reduce intrusions from unwanted memories. Specifically, we report two experiments testing whether people who have experienced more traumatic events are better at inhibiting memories compared with individuals who were largely spared from such experiences. We asked participants to perform a version of the Think/No-Think (hereinafter, TNT) task (Anderson & Green, 2001) that incorporated emotionally neutral and negative items. The task requires participants to attend to reminders of previously acquired associations involving those items. For each reminder, they are cued either to retrieve the associated memory (Think trials) or to instead suppress its retrieval (No-Think trials). Repeatedly suppressing retrieval impairs retention of the associated memory, a phenomenon known as suppression-induced forgetting (Anderson & Green, 2001; see Anderson & Hanslmayr, 2014, for a review). Retrieving memories, in contrast, enhances later recall of the associates. Notably, comparable fronto-parietal control regions are engaged during suppression of neutral and negative content (e.g., Anderson et al., 2004; Butler & James, 2010; Depue, Curran, & Banich, 2007; Gagnepain, Hulbert, & Anderson, 2017; see Anderson & Hanslmayr, 2014, for a review), and successful engagement of these regions to suppress retrieval also reduces negative affect associated with unpleasant images (Gagnepain et al., 2017).

In our adaptation of the TNT task, participants were first trained on word pairs consisting of neutral cues (e.g., *street*) and either neutral (*violin*) or unpleasant response words (*corpse*). Participants then were instructed to retrieve or suppress the associated response 0 (Baseline), one or 16 times during the TNT phase. Following this manipulation, we measured the accessibility of all of the response words using two surprise cued-recall tests, the order of which was counterbalanced across participants: the “same-probe” test and the “independent-probe” test (Anderson & Green, 2001). Whereas the same-probe test (hereinafter the SP test) measured accessibility by using the same cues originally studied with the response, the independent-probe test (hereinafter, the IP test) instead used extralist semantic cues (e.g., *anatomy c___*). This latter test provides a purer assay of the aftereffects of inhibitory processes on the suppressed response because it sidesteps the original cue-target association (Anderson & Green, 2001). Indeed, the SP test likely mixes the contributions of persisting memory inhibition with associative interference (Anderson & Huddleston, 2011; Anderson & Levy, 2009; Noreen & de Fockert, 2017; Wang, Cao, Zhu, Cai, & Wu, 2015), making it harder to isolate individual differences in inhibitory processes using this test of suppression-induced forgetting (Anderson & Levy, 2007). Item-specific cuing tests, such as our IP measure, are more sensitive to individual differences in inhibition (e.g., Anderson, Reinholz, Kuhl, & Mayr, 2011; for related findings, see Schilling, Storm, & Anderson, 2014). Suppression-induced forgetting has regularly been observed using IP tests for a variety of stimuli (see Anderson & Hanslmayr, 2014; Anderson & Huddleston, 2011, for reviews), including emotional materials (e.g., see Lambert, Good, & Kirk, 2010; Murray, Anderson, & Kensinger, 2015; Murray, Muscatell, & Kensinger, 2011).

To determine whether self-reported trauma is related to individual differences in suppression-induced forgetting, we asked college-aged participants to estimate the number of traumas they experienced prior to the age of 18 using a survey that addressed a wide range of trauma types (Goldberg & Freyd, 2006). We then examined whether individuals reporting more traumatic experiences demonstrated greater suppression-induced forgetting on the final tests of the TNT paradigm for both negative and neutral target items. Given the similarity in the brain systems engaged to suppress retrieval of negative and neutral content in prior work (Anderson et al., 2004; Depue et al., 2007; Gagnepain et al., 2017), we predicted that any advantage in suppression-induced forgetting should arise regardless of the valence of suppressed content. Indeed, to the extent that traumatic experience augments memory control abilities generally, the advantage should extend beyond negative materials. Nevertheless, it is possible that there may be an additional adaptive benefit for materials that give rise to a negative affective context more closely matching that which motivated suppression practice outside of the laboratory. To rigorously compare the relative magnitude of suppression-induced forgetting across valences, we (a) carefully matched negative and neutral response words on length, frequency, concreteness, and other dimensions that are sometimes confounded with valence manipulations (see Method) and (b) ensured that the same reminder cues paired with neutral targets were also paired with negative targets (across participants). Finally, given prior evidence that item-specific, cue-independent measures of forgetting are more sensitive to individual differences in inhibitory control (Anderson et al., 2011; Schilling et al., 2014), we predicted that the influence of prior trauma history on suppression-induced forgetting may be especially evident on our IP test of cue-independent forgetting.

## Experiment 1

In Experiment 1, we defined the lower- and higher-trauma groups based on a postexperimentally administered traumatic experiences survey (Goldberg & Freyd, 2006). The survey asked participants to estimate the frequency of a range of traumatic incidents (e.g., witnessing or experiencing accidents, natural disasters, violence, sexual assault/abuse, emotional abuse, and the death of significant individuals) separately for the periods before age 18 and afterward. We divided our sample into equal groups reporting lower and higher levels of total trauma events prior to the age of 18, while holding our stimulus and test order counterbalancing factors constant. We sought to establish two groups that were treated identically during the experiment, notwithstanding their differing levels of self-reported trauma. If a greater history of trauma provides a natural opportunity to practice retrieval suppression, and if these experiences yield a generalized suppression skill, we should find greater suppression-induced forgetting in our higher-trauma group.

### Method

#### Participants

Participants were undergraduates (*N* = 48) receiving credit for a course requirement. The stopping point for data collection was preselected based on previous research using a similar design (Anderson & Green, 2001) and was constrained by counterbalancing (there were 24 unique cells after combining our stimulus and test order counterbalancing factors; see Materials). An additional five participants were run but were excluded and will not be considered further on the basis of their inability to reach criterion for initial learning (*n* = 2), failure to comply with instructions (*n* = 2), or history of brain damage (*n* = 1). These exclusion criteria were based on long-standing lab protocols (see, e.g., Anderson et al., 2004, 2011) and supported by empirical evidence (e.g., Hertel & Calcaterra, 2005). All participants provided written informed consent in accordance with the protocol approved by the University of Oregon’s Institutional Review Board.

#### Materials

##### Stimuli

We predicted that more extensive experience inhibiting unwanted memories would lead to improved suppression ability on material that was not necessarily related to the original trauma (cf., Catarino et al., 2015). As such, a single set of 60 critical word pairs (e.g., *street-violin*; *trunk-corpse*) and six fillers was constructed, each pair being composed of a neutral cue word and either a neutral or negative response word. The full stimulus set can be found in Table S3 in the online supplemental materials.

Cue words were counterbalanced across participants, such that each neutral cue appeared equally often with neutral and negative response words (e.g., one set of participants would receive the above pairings, whereas another set would study *trunk-violin* and *street-corpse*). The cues were selected to be moderately relatable to their target responses but unrelated to any other words in the stimulus set.

Negative response words were defined as having a valence rating of less than 3.00, whereas the valence ratings for neutral words fell between 4.02–5.98 according to the Affective Norms for English Words (ANEW; Bradley & Lang, 1999). In addition to the deliberate difference in valence (*M*_*neutral*_ = 5.61, *SD*_neutral_ = .53; *M*_*negative*_ = 2.21, *SD*_negative_ = .51), negative responses were also associated with significantly greater arousal and reduced dominance, *p* < .001 in all cases. In the instances for which norming data were unavailable, an independent sample of 10 raters used comparable methodologies to determine the relevant values. Response members of word pairs were carefully selected so that, across item sets (negative vs. neutral items), they did not differ on any of the following dimensions: word-length, syllables, word frequency, concreteness, or associative set size (Nelson, McEvoy, & Schreiber, 2004), *p* > .24 in all cases. Special care was taken to avoid strong preexisting associations between any two responses.

Independent probes for all of the response words were constructed by selecting other words that were highly related to the target response but unrelated to the original cues (e.g., *lessons-vi__* for *violin*) according to norms (Nelson et al., 2004). Unlike in most studies using independent probes (see Anderson & Hanslmayr, 2014, for a review), we provided a two-letter stem cue (*vi__* in the above example) instead of a single letter stem. This change in lexical information was motivated by our use, in this study, of associative independent probes (e.g., *lessons-* for *violin*) instead of categorical ones (e.g., *meat-* for *beef*). The change in cue type was necessitated by our manipulation of response valence: Because we required 30 neutral and 30 negatively valenced response words, it was challenging to identify enough distinct semantic categories to serve as independent probes in each grouping. Because of this, we cued with semantic associates instead of categories. Out of concern that associations may be too weak as cues, we provided additional cuing support by increasing the lexical information provided by the stem.

##### Traumatic experiences survey

We administered the Brief Betrayal-Trauma Survey (BBTS; Goldberg & Freyd, 2006) after participants completed the TNT task. The 12-item survey asked participants to estimate the frequency of traumatic incidents separately for the periods before age 18 and afterward. Because the mean age of participants in Experiment 1 was only 20.35 years (*SD* = 2.21), we operationalized trauma history as the average frequency score based on events that happened before age 18, disregarding type of trauma. Specifically, these average scores were derived from participants’ best estimate of the number of instances of each type of trauma assessed using a 6-point scale for each item on the survey [0 = *never*; 1 = *one time*; 2 = *two to five times*; 3 = *six to 20 times*; 4 = *21 to 100 times*; 5 = *more than 100 times*]. See Table S1 in the online supplemental materials for descriptive statistics and further demographic information.

#### Procedure

Participants initially viewed each of the word pairs for 6 s. After this study phase, we drilled participants on the pairs. We presented each cue and asked participants to vocally respond with the associated response word within a 4-s time window. We provided corrective feedback after each trial. We gave participants up to three cycles to correctly recall at least 50% of the items, a training threshold representative of many studies (e.g., Anderson & Green, 2001; Anderson et al., 2004; Benoit & Anderson, 2012; van Schie & Anderson, 2017).

After reaching learning criterion, participants entered the TNT phase, in which we instructed them to say the associated response word to any cue word presented in green as fast as possible (feedback would be presented if they failed to respond out loud). In contrast to these Think trials, if a cue word appeared in red (indicating a No-Think trial), they were to avoid both thinking about *and* saying the response word for the same 4-s trial duration. In all cases, participants were to direct their eyes and attention to the cue word. Cue words appeared consistently in either green or red 0 (Baseline), 1, or 16 times during the phase. The counterbalancing of word pairs across these six conditions (0, 1, or 16 repetitions in either the Think or No-Think instruction conditions) crossed with the two possible response pairings described earlier (neutral and negative valence), produced 12 counterbalancing cells to achieve full balancing of stimulus materials across conditions.

Two surprise recall tests were then administered for all of the learned responses (Anderson & Green, 2001). The same probe (SP) test presented participants with the original cue words, for which they had up to 4 s to say the associated response aloud. The independent probe (IP) test was structured similarly, except that the probe consisted of a novel semantic cue and a word stem. The two tests were administered in a counterbalanced order across participants. Combining this test order factor with the 12 stimulus-counterbalancing levels discussed above yielded a total of 24 unique counterbalancing combinations. For both the SP and IP tests, we encouraged participants to provide the correct answer as quickly as possible for every probe, regardless of the instructions during the TNT phase.

After the final memory tests, participants rated each word from the stimulus set and a random selection of 30 positive words in terms of valence using a method akin to the ANEW (Bradley & Lang, 1999). A postexperimental questionnaire assessing compliance was then administered prior to the Beck Depression Inventory (see Table S1 for BDI scores; Beck, Steer, & Carbin, 1988). Finally, we asked participants to complete Goldberg and Freyd’s (2006) traumatic experiences survey, which was used to establish their group status. Given that 24 participants were required to obtain even counterbalancing, we ran 48 participants, to enable construction of two groups, matched perfectly for counterbalancing, but differing in reported trauma.

#### Analysis approach

In analyzing the final test recall data, we first examined suppression-induced forgetting and retrieval-induced facilitation effects across test types (i.e., aggregated over the SP and IP tests) to characterize the general influence of our instruction manipulation on recall, irrespective of the nature of the final test cue. We then followed these analyses with tests of possible interactions by test type to determine whether the effects of interest generalized over different retrieval cues used to assess performance. If suppression-induced forgetting is cue-independent, for example, we would expect it to emerge in response to both types of test cues. If a significant interaction with test type was observed, we characterized these effects separately for each test type to isolate the nature of the interaction. If an overall effect was significant but no interaction observed, we interpreted this to suggest that test type did not moderate the overall effect, consistent with cue-independence.

We analyzed the relation between trauma experience and inhibitory control over memory via a group analysis (see later section entitled “Integrated Analyses of Experiments 1 and 2” for a complementary analysis using robust correlation). We divided our sample into equal groups reporting lower and higher levels of trauma, while holding our stimulus and test order counterbalancing factors constant. Thus, lower- and higher-trauma groups were established via a median split based on participants’ trauma scores, separately within each of our 24 counterbalancing conditions. By combining the lower-trauma halves of each of these splits together, we formed a fully counterbalanced sample that had lower trauma scores than did the corresponding higher-trauma group, which was similarly composed by combining the higher-trauma halves of these splits (for other examples of this approach, see, e.g., Anderson et al., 2004; Hanslmayr, Leipold, Pastötter, & Bäuml, 2009). In cases for which participants had the same trauma frequency (of which there were two), ties were broken based on the number of traumas reported after the age of 18 or, in a single case, a coin toss.^1^

One disadvantage of our group analysis approach is that it runs the risk of reducing the difference in level of trauma across the higher- and lower-trauma groups. By conducting the median split within each counterbalancing group, for example, it is possible for the “higher” trauma participants in one split to have lower levels of trauma than “lower” trauma groups in a different split for another counterbalancing group. Thus, by insisting on the constraint that the groups be matched across all counterbalancing dimensions, the trauma scores of the two groups may have some overlap. To determine whether this issue compromised how strongly our lower- and higher-trauma groups differed in their level of trauma, we conducted a check on the quality of our split. To do this, we compared the trauma levels in our carefully matched median split to the trauma levels that would have been obtained had we simply performed a median split on the entire sample of 48, disregarding counterbalancing. This latter version of the split revealed a difference in average trauma scores across the groups that broadly matched that of the groups formed using the original procedure (compare Table S1 with Table S2), suggesting that our matching procedure did little to compromise how strongly the higher- and lower-trauma groups differed.

### Results

#### Training phase performance

Participants took 1.88 (*SD* = 0.70) cycles on average to reach criterion (averaging 41 out of 66 items correct in their final cycle, *SD* = 5.88), discounting five participants (three lower-trauma and two higher-trauma participants) for whom the criterion data were not retained in error. Neither the number of cycles to reach criterion, *t*(41) = 0.63, *p* = .535, nor the number of correct items in the last cycle, *t*(41) = −0.07, *p* = .946, differed significantly across higher- and lower-trauma groups (two-tailed tests).

#### Final test phase performance

By crossing the instruction factor (Think vs. No-Think) with repetition (0 vs. 16), our experiment had two identical Baseline (0 repetitions) cells. Because these cells should not meaningfully differ, we combined them for ANOVAs that focused on the suppression-induced forgetting and retrieval-induced facilitation effects. The ANOVA addressing the overall memory control effect (Think vs. No-Think), however, necessitated separation of these baselines (e.g., testing the interaction of repetition by instruction).^2^

#### Overall memory control effects

**Figure 1 fig1:**
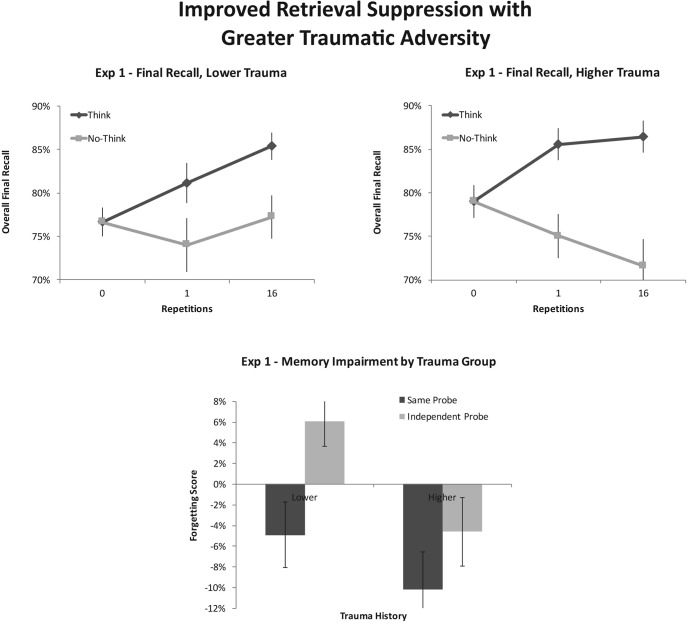
Final test results by trauma group in Experiment 1. The top panels present aggregate final recall (averaged across same-probe [SP] and independent-probe [IP] measures) scores relative to Baseline performance for Think and No-Think items as a function of repetition. Being repeatedly exposed to reminders facilitated Think items in a way not observed for No-Think items in either trauma group. Yet, only the higher-trauma group (top right panel) exhibited significant below-Baseline forgetting of No-Think items as a result of retrieval suppression. The bottom panel depicts suppression-induced forgetting (16 No-Think repetitions – 0 repetition Baseline) separately on SP and IP tests of forgetting. Negative values represent suppression-induced forgetting as a result of previous suppression attempts, whereas positive values represent suppression-induced facilitation. Error bars reflect *SE*s.

Participants showed robust control over the retrieval process, as reflected by the interaction of instruction with repetition on final test recall, *F*(1, 24) = 20.86, *p* < .001, η_*p*_^2^ = .465. Both lower-trauma participants, *F*(1, 24) = 3.17, *p* = .088, η_*p*_^2^ = .117, and higher-trauma participants, *F*(1, 24) = 21.90, *p* < .001, η_*p*_^2^ = .477, showed evidence for control effects, as can be seen in Figure 1. This recall benefit for Think items relative to No-Think items indicates that participants could, at a minimum, stop the retrieval process from occurring on No-Think trials often enough to preempt the strengthening/facilitation that retrieved Think items usually exhibit. It does not, however, address the separate effects of retrieval-induced facilitation (specifically, Think recall relative to Baseline) or suppression-induced forgetting (specifically, No-Think recall relative to Baseline), which we discuss next.[Fig-anchor fig1]

#### Retrieval-induced facilitation

Consistent with prior work, we found that final recall performance for Think items was reliably better after 16 retrieval attempts than after none (Baseline), *F*(1, 24) = 61.79, *p* < .001, η_*p*_^2^ = .720. This effect did not interact with valence, *F* < 1. Importantly, the facilitation did not differ reliably across trauma groups, *F* < 1, despite a small numerical tendency for higher-trauma participants to show less facilitation. Thus, attending to Think items produced retention benefits that were statistically similar for the two trauma groups. This similarity of facilitation across higher- and lower-trauma groups was true irrespective of target item valence, as reflected in a nonsignificant trauma History × Repetition × Valence (neutral vs. negative) interaction, *F* < 1.

Facilitation was, however, greater when Think items were tested on the SP compared with the IP test, *F*(1, 24) = 85.10, *p* < .001, η_*p*_^2^ = .780. In particular, whereas the SP test showed robust facilitation of Think items, *F*(1, 24) = 151.45, *p* < .001, η_*p*_^2^ = .863, the IP test showed no evidence of facilitation, *F* < 1. This attenuated facilitation on the IP test is consistently observed in the Think/No-Think paradigm (see Anderson & Huddleston, 2011, for a meta-analysis of 1300 participants tested with IPs) and indicates that the benefits of repeated retrieval on later retention are primarily associative and cue-specific.

#### Suppression-induced forgetting

Next, we examined whether suppressing the retrieval process impaired final test performance for No-Think items relative to Baseline performance. To estimate suppression, we compared recall of No-Think response words after 0 suppression attempts (Baseline) to performance after 16 suppression attempts. We first tested for suppression-induced forgetting, collapsed over type of test and valence, to assess the effect of suppression on overall retention. The group, as a whole, showed better recall in the Baseline condition than after retrieval suppression, although this effect was marginally significant, *F*(1, 24) = 4.06, *p* = .055, η_*p*_^2^ = .145. Thus, independent of trauma history, suppressing retrieval tended to impair overall retention of suppressed items in a manner largely consistent with prior work. The counterbalanced order in which the two constituent tests were administered did not affect this conclusion, as reflected in a nonsignificant interaction of test Order × Repetition, *F*(1, 48) = 1.47, *p* = .232, η_*p*_^2^ = .030. Importantly, the repetition effect did not interact with the emotional valence of the suppression target, *F* < 1, indicating that the effect was comparable for both neutral and negative items.

Of key interest, however, was whether participants who reported having lived through more traumatic experiences differed in how well they contended with unwanted memories. Strikingly, we found that, whereas participants with greater experience with trauma displayed significant forgetting of No-Think items (Baseline minus No-Think recall = 80% − 72%, yielding an 8% suppression-induced forgetting effect), *F*(1, 24) = 9.62, *p* = .005, η_*p*_^2^ = .286, the lower-trauma group displayed no reliable evidence of this ability to forget, (Baseline minus No-Think recall = 77% − 77%, yielding a 0% effect), *F* < 1. This difference in suppression-induced forgetting was significant, as reflected by an interaction of repetition and trauma, *F*(1, 24) = 5.62, *p* = .026, η_*p*_^2^ = .190. Importantly, this apparent trauma-history advantage did not interact reliably with the emotional valence of the target material being suppressed, *F* < 1, suggesting that it reflects a generalized skill of suppression, regardless of valence. Thus, more extensive trauma exposure was associated with an enhanced ability to suppress unwanted memories.

#### Variation in suppression-induced forgetting by test type

To determine whether suppression impaired access to unwanted memories in a cue-independent manner (Anderson & Green, 2001), we tested whether suppression-induced forgetting varied across our SP and IP tests. Contrary to our expectation, we observed a significant interaction of Repetition × Test type, *F*(1, 24) = 11.28, *p* = .003, η_*p*_^2^ = .320, with significant suppression-induced forgetting on the SP test, *F*(1, 24) = 11.53, *p* = .002, η_*p*_^2^ = .325, but no reliable forgetting on the IP test, *F* < 1. This variation in suppression-induced forgetting across test type did not interact with the valence of target items, *F* < 1.

Despite the lack of an overall effect of suppression-induced forgetting on the IP test, this finding does not address the origins of trauma-history advantage. To the extent that greater suppression-induced forgetting for higher-trauma participants reflects an enhanced ability to inhibit memories, the suppression advantage should generalize to IPs. To test this, we examined the interaction of Repetition × Trauma × Test type. Consistent with this generalization, the trauma-history advantage in suppression-induced forgetting did not interact with test type, *F*(1, 24) = 1.18, *p* = .289, η_*p*_^2^ = .047. As can be seen in Figure 1, participants with lower trauma showed reliable suppression-induced *facilitation* on the IP test, *F*(1, 24) = 4.88, *p* = .037, η_*p*_^2^ = .169, whereas higher-trauma participants show a weak trend toward suppression-induced *forgetting*, *F*(1, 24) = 2.76, *p* = .109, η_*p*_^2^ = .103. The foregoing findings suggest that the suppression-induced forgetting advantage for those reporting higher levels of trauma might arise, in part, from greater engagement of inhibition to stop retrieval.

### Discussion

Experiment 1 indicates that people who report having greater experience with traumatic events may, indeed, be better at suppressing the retrieval of unwanted memories, even in our simple laboratory task. As predicted, participants in our higher-trauma group exhibited greater suppression-induced forgetting than did participants in our lower-trauma group. Interestingly, although the latter group could likely prevent retrieval from happening (as suggested by the lack of an overall positive benefit of being repeatedly exposed to reminders for No-Think items), they showed no evidence of an ability to forget the items that they had tried to suppress. The superior forgetting exhibited by our higher-trauma group did not vary across negative or neutrally valenced materials, consistent with the possibility that the mechanism underlying the retrieval-suppression advantage is not tied to aversive experiences, per se, but rather to controlling mnemonic content in general. The trauma-history advantage also did not vary with test type, with similar benefits on the SP and IP tests, consistent with the possible contribution of improved inhibitory control over memory. Indeed, lower-trauma participants showed a reversal of suppression on the IP test, indicating that their forgetting is cue-dependent.

In Experiment 1, overall suppression-induced forgetting did not generalize to the IP test. This contrasts with other, similarly conducted experiments showing comparable effects on SP and IP tests (see Anderson & Hanslmayr, 2014, for a review; see also Wang et al., 2015). One possibility is that reduced IP forgetting may be linked to the additional lexical information provided to subjects: Unlike in most other TNT studies, which most commonly use IP cues with a single letter stem (e.g., Anderson & Green, 2001; Anderson et al., 2004) or no stem (Wang et al., 2015), we provided two letters. Moreover, the IPs were associative (e.g., *anatomy*-*co__* for *corpse* and *bag*-*ga__* for *garbage*) rather than categorical (e.g., *meat*-*be__* for *beef*). Associative cues might have been more difficult, prompting greater reliance on a lexical search strategy in response to the expanded letter stem. Evidence suggests that indirect word fragment completion tasks are less sensitive to suppression-induced forgetting (Angello, Storm, & Smith, 2015). If so, then our IP measure of inhibition may have been diluted by nonepisodic lexical retrieval.

Experiment 1 did not clearly support our prediction that IP tests would be more sensitive to the hypothesized differences in inhibitory control. Specifically, we predicted that the difference between lower- and higher-trauma groups in suppression-induced forgetting would be larger on the IP test. Although there was a trend in that direction (higher-trauma participants displayed an 11% average suppression-induced forgetting effect on the SP test, compared with a 5% effect displayed by lower-trauma participants—resulting in a 6 percentage-point forgetting advantage for higher-trauma participants; on the IP test, by contrast, higher-trauma participants displayed a 5% average suppression-induced forgetting effect, compared with a −6% effect displayed by lower-trauma participants—resulting in an 11 percentage-point forgetting advantage for higher-trauma participants; see Table 1), this interaction was not significant. The predicted sensitivity of the IP test may yet emerge if the contributions of lexical retrieval are controlled.[Table-anchor tbl1]

Although the foregoing results broadly support a trauma-history advantage in suppression-induced forgetting, it is important to consider whether, our findings truly reflect a greater capacity to forget, or instead the contributions of experimenter/participant biases. Although unlikely, by administering the traumatic experiences survey at the end of the experiment, the experimenters could, in principle, have been unintentionally influenced in their scoring of the data (e.g., to the extent that any responses were ambiguous) by their knowledge of a participant’s trauma status. Alternatively, when participants attempted to suppress the retrieval of unwanted memories during our task, they may have formed opinions about the purpose of the experiment that led them to alter their responses to the traumatic experiences survey in some fashion. Some participants also might have been tempted to withhold correctly recalled responses based on these demands. Although largely excluded as an account of suppression-induced forgetting in several experiments previously (Anderson & Green, 2001), it is important to consider whether, in the current study, such biases could play a role. We address these possibilities, along with the foregoing issues about cue-independent forgetting, in Experiment 2.

## Experiment 2

Experiment 2 sought to replicate the findings of Experiment 1 but with even more rigorous procedural controls. These controls were designed to ensure that (a) neither experimenter nor participant bias contaminated any evidence for trauma-related improvements in memory control, and (b) participants were suitably motivated on the final test to recall the items they had suppressed. Toward these ends, instead of administering the traumatic experiences survey postexperimentally, we administered it in a multilaboratory battery weeks (or longer) before the experiment, in an independent prescreening session online. This procedure ensured a clean separation of higher- and lower-trauma groups by their reported level of traumatic experience. Moreover, it disguised any relationship between our experiment and participants’ trauma history, so that participants’ responses on the survey would neither (a) be influenced by taking part in the TNT task, nor (b) be known to experimenters administering the procedure or scoring the data. To ensure the latter point, we also used a blinding procedure whereby the laboratory coordinator assigned experimenters to participants in a manner that did not identify participants’ trauma status. Moreover, during the final tests, we tried to ensure that participants always reported a recalled response, if they could. We modified the test instructions to strongly emphasize the need to provide answers to all items, if possible, even if participants were unsure of their answers. We also offered monetary incentives for each critical memory item correctly recalled (cf. Anderson & Green, 2001) to motivate participants to report every answer. Finally, Experiment 2 also modified the IP test by reducing the number of letters in the stem cue from two to one to increase sensitivity of the test to inhibition. As in Experiment 1, we predicted that the pattern of greater forgetting for higher trauma participants should generalize over cues, and so, not vary as a function of the type of test.

### Method

#### Participants

We selected participants in the higher- or the lower-trauma groups on the basis of their scores on Goldberg and Freyd’s (2006) traumatic experiences survey, as in Experiment 1. However, this survey was administered with other measures across online prescreening sessions for introductory psychology classes (*N* = 512 over two semesters) as part of a multilab effort. From this, potentially eligible recruits were invited to participate in what was ostensibly an independent study, without foreknowledge that their self-reported trauma scores for events occurring prior to the age of 18 defined their assignment to either the lower- or higher-trauma groups within each one of our counterbalancing conditions.

Forty-eight undergraduates participated in exchange for course credit (and a potential monetary bonus), run by an experimenter who was blind as to their trauma history (see below). We ran an additional nine participants to replace subjects who were excluded because of an inability to reach the initial learning criterion (*n* = 7), failure to comply with instructions (*n* = 1), or prior exposure to the TNT paradigm (*n* = 1). All participants provided written informed consent in accordance with the protocol approved by the University of Oregon’s Institutional Review Board.

#### Materials and procedure

The materials and procedure were identical to those in Experiment 1, with the following exceptions.

##### Final test

We reduced the length of the word stem used for IPs to be a single letter instead of two letters as was used in Experiment 1, to increase sensitivity to inhibition. In addition, the final test instructions for Experiment 2 put additional emphasis on the importance of recalling as many of the responses as possible, even if participants were unsure of their answers. To emphasize this point further and to facilitate motivation, we offered a monetary reward of 20 cents for any correct response (provided on at least one of the two test types) for a predetermined subset of final test items (cf. Anderson & Green, 2001). Participants were informed that they could earn up to $4.00 for providing correct responses on unspecified “critical items,” which happened to consist of the five No-Think responses each from their 1-repetition neutral, 1-repetition negative, 16-repetition neutral, and 16-repetition negative counterbalanced sets.

##### Blinding procedures

To ensure that experimenters were not biased in the manner in which they administered the TNT task to participants in the higher- or lower-trauma groups, we adopted a blinding procedure in which experimenters had no knowledge of the group that participants were in. A laboratory coordinator randomly assigned participants to experimenters and did not label participant materials in a way that indicated group status. Finally, to ensure that data coding was unbiased, experimenters remained blinded to participants’ group status while scoring recall performance.

### Results

#### Training phase performance

Participants took 1.75 (*SD* = 0.70) cycles on average to reach criterion (averaging 41 of 66 items correct in their final cycle, *SD* = 6.04). Neither the number of cycles to reach criterion, *t*(46) = 1.25, *p* = .219, nor the number of correct items in the last cycle *t*(46) = 0.55, *p* = .588, differed significantly across higher- and lower-trauma groups (two-tailed tests).

#### Final test phase performance

##### Overall memory control effects

**Figure 2 fig2:**
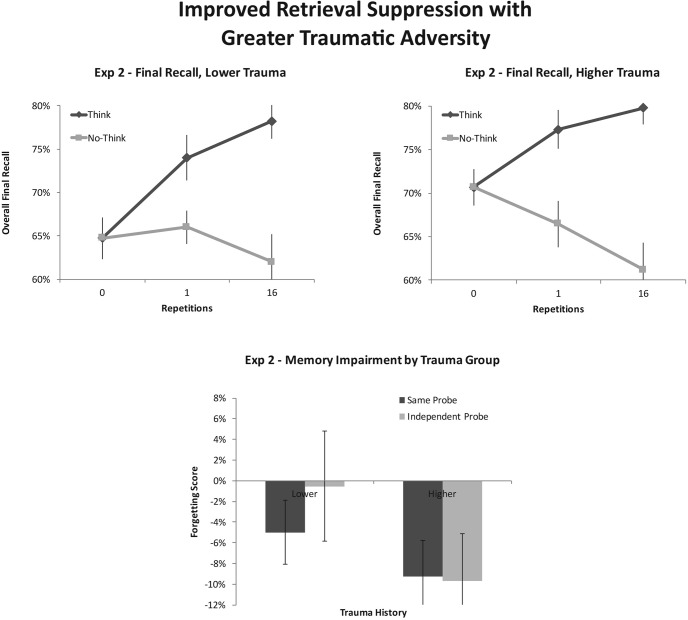
Final test results by trauma group in Experiment 2. The top panels present aggregate final recall (averaged across same-probe [SP] and independent-probe [IP] measures) scores relative to Baseline performance for Think and No-Think items as a function of repetition. Being repeatedly exposed to reminders facilitated Think items in a way not observed for No-Think items in either trauma group. Only the higher-trauma group (top right panel) exhibited significant below-Baseline forgetting of No-Think items as a result of retrieval suppression. The bottom panel depicts suppression-induced forgetting (16 No-Think repetitions – 0 repetition Baseline) separately on SP and IP tests of forgetting. Negative values represent suppression-induced forgetting as a result of previous suppression attempts. Error bars reflect *SE*s.

Participants showed robust control over the retrieval process, as reflected by the interaction of instruction with repetition, *F*(1, 24) = 30.30, *p* < .001, η_*p*_^2^ = .588. This overall memory control effect did not vary by trauma group, *F* < 1. Both lower-trauma participants, and higher-trauma participants, showed evidence for control effects, as can be seen in Figure 2 and Table 1. This indicates that, as in Experiment 1, participants could, at a minimum, stop the retrieval process from occurring on No-Think trials often enough to preempt the strengthening/facilitation that retrieved items usually exhibit. We next turn to the separate effects of retrieval-induced facilitation and suppression-induced forgetting.[Fig-anchor fig2]

##### Retrieval-induced facilitation

The facilitation effects in Experiment 2 replicated those observed in Experiment 1 in nearly every respect. Final recall performance for Think items was reliably greater after 16 retrieval attempts than after none (Baseline), *F*(1, 24) = 48.98, *p* < .001, η_*p*_^2^ = .671. This effect that did not interact with valence, *F* < 1. Importantly, as in Experiment 1, the facilitation did not differ across the trauma groups, *F*(1, 24) = 1.83, *p* = .188, η_*p*_^2^ = .071. Thus, retrieving Think items produced retention benefits that were statistically similar for the two trauma groups. This similarity of facilitation across groups was true irrespective of target item valence, as reflected by a nonsignificant Repetition × Trauma × Valence interaction, *F*(1, 24) = 1.97, *p* = .173, η_*p*_^2^ = .076.

As in Experiment 1, facilitation was greater when we tested Think items on the SP compared with the IP test, *F*(1, 24) = 11.57, *p* = .002, η_*p*_^2^ = .325. In particular, whereas the SP test showed robust facilitation of Think items, *F*(1, 24) = 98.61, *p* < .001, η_*p*_^2^ = .804, the IP test showed reliable, but quantitatively less facilitation, *F*(1, 24) = 5.06, *p* = .034, η_*p*_^2^ = .174. The latter finding deviates from Experiment 1, which found no evidence of facilitation on the IP test, perhaps indicating that reducing letter stem cues in Experiment 2 improved sensitivity to facilitation. Nevertheless, the facilitation was less pronounced on the IP test, which is consistently observed in the Think/No-Think task (Anderson & Huddleston, 2011).

##### Suppression-induced forgetting

As with facilitation, the overall suppression-induced forgetting findings from Experiment 2 replicated Experiment 1. Collapsing over trauma group, test type, and valence, the sample as a whole showed robust suppression-induced forgetting, with better recall in the Baseline condition (0 repetitions) than after 16 retrieval suppression repetitions, *F*(1, 24) = 8.52, *p* = .008, η_*p*_^2^ = .262. Thus, independent of trauma history, suppressing retrieval impaired overall retention of No-Think items in a manner consistent with Experiment 1 and prior work. The counterbalanced order in which we administered the two constituent tests did not affect this conclusion, as reflected by a nonsignificant interaction of test order and repetition, *F*(1, 24) = 1.06, *p* = .308, η_*p*_^2^ = .024. Importantly, the suppression-induced forgetting effect showed no interaction with the valence of the suppression target, *F* < 1, demonstrating that the effect was comparable for both neutral and negative items.

Of key interest, however, was whether higher-trauma participants would show superior suppression-induced forgetting, as they had in Experiment 1, despite the array of procedural controls introduced in Experiment 2. Critically, whereas participants with higher trauma displayed robust forgetting of No-Think items (Baseline minus No-Think recall = 71% − 62%, a 9% effect), *F*(1, 24) = 10.22, *p* = .004, η_*p*_^2^ = .299, the lower-trauma group, in contrast, displayed no reliable evidence of an ability to forget No-Think items, (Baseline minus No-Think recall = 64.5% − 62% = a 2.5% effect), *F*(1, 24) = 0.87, *p* = .361, η_*p*_^2^ = .035. The interaction of trauma Group × Repetition, however, did not quite reach significance in this sample, *F*(1, 24) = 2.57, *p* = .122, η_*p*_^2^ = .097. Nevertheless, the pattern of suppression observed across the higher- and lower-trauma groups is both qualitatively and quantitatively similar to that observed in Experiment 1 (see section entitled “Integrated Analyses of Experiments 1 and 2” for a formal statistical comparison of these patterns). Importantly, this apparent trauma-history advantage did not interact reliably with the emotional valence of the target material being suppressed, *F*(1, 24) = 1.64, *p* = .213, η_*p*_^2^ = .064, suggesting that this effect reflects a generalized skill at suppression, regardless of valence.

##### Variation in suppression-induced forgetting by test type

Unlike in Experiment 1, suppression-induced forgetting did not vary reliably across our SP and IP tests, *F* < 1, with similar forgetting on the SP test (Baseline minus No-Think = 80% − 73% = 7%), and on the IP test (Baseline minus No-Think = 55% − 50% = 5%). Thus, suppression-induced forgetting generalized over cues, a pattern that did not interact with the valence of target items, *F* < 1. These findings suggest that participants in Experiment 1 may have been relying on a lexically focused retrieval strategy, a possibility consistent with the fact that overall IP recall performance fell precipitously from 76% in Experiment 1 to 57% in Experiment 2 (a 19% reduction), a drop that was highly significant, *F*(1, 48) = 98.44, *p* < .001, η_*p*_^2^ = .672.

Next, we examined whether the greater suppression-induced forgetting for higher-trauma participants reflected an enhanced ability to inhibit unwanted memories. If so, we would expect the trauma-history advantage to generalize to IPs, as it did in Experiment 1. To test this, we examined the interaction of Repetition × Trauma × Test type. Consistent with this possibility, the trauma-history advantage in suppression-induced forgetting showed no evidence of interacting with test type, *F* < 1. This reflects the fact that participants with lower reported trauma showed weaker suppression-induced forgetting than higher-trauma participants (Baseline minus No-Think recall) on both the SP test (lower trauma: 78% − 73% = 5%; higher trauma: 82% − 73% = 9%) and the IP test (lower trauma: 51% − 51% = 0%; higher trauma: 59% − 50% = 9%; see Figure 2). As in Experiment 1, the difference in SIF across higher- and lower-trauma groups was numerically smaller on the SP test (9% − 5% = 4%) than on the IP test (9% − 0% = 9%), consistent with the possibility that the IP test is more sensitive to differences in inhibitory function. However, as detailed above, this interaction was not reliable. Overall, these findings suggest that the suppression-induced forgetting advantage for higher-trauma participants arises, in part, from better engagement of inhibition.

### Discussion

Experiment 2 suggests that the trauma-history advantage in suppression-induced forgetting does not arise from participant or experimenter bias or from participants not being motivated to recall or report suppressed items. In Experiment 1, bias could have influenced the data in several ways: awareness of participants’ trauma status (as reported on the postexperimental questionnaire) could have influenced how experimenters scored the data; or participants might have altered their trauma history reporting after having participated in a task involving suppression; or participants might have guessed the purpose of the study (to find evidence for forgetting) and then deliberately withheld correctly recalled No-Think items on the test. By collecting trauma-history data in a separate and seemingly unrelated session weeks earlier, by adopting rigorous blinding procedures, and by strongly encouraging participants to report all recalled responses (and adding monetary incentives), Experiment 2 bolsters confidence that the trauma-history advantage reflects a genuine difference in forgetting of the memories people tried not to think about. This advantage did not vary as a function of the valence of the suppressed items, suggesting that it reflects a generalized enhancement of the suppression process. Experiment 2 also replicated the finding that the trauma-history advantage in suppression-induced forgetting did not vary by test type, demonstrating that the superior forgetting generalized across cues. This finding is consistent with the possibility that the advantage reflects better inhibitory control in the higher-trauma group.

## Integrated Analyses of Experiments 1 and 2

To explore the relation of trauma history and suppression-induced forgetting with greater statistical power, we combined the data from our two similar experiments. In this section, we report this analysis, which includes the same factors as in the experiment-specific analyses, but with an additional between-subjects experiment factor. This analysis enables us to establish, in a large sample, the overall robustness of patterns observed in individual experiments and to assess the reliability of any apparent differences across studies. We also report a complementary set of robust and partial correlation analyses based on the combined sample to examine whether a continuous relationship exists between the frequency of trauma and suppression-induced forgetting, as well as to assess the contributions of potential confounding variables, such as depression or Baseline performance.

### Results

#### Overall memory control effects

Participants showed extremely robust control over the retrieval process, as reflected by the interaction of instruction (Think vs. No-Think) with repetition (0 vs. 16), *F*(1, 48) = 55.10, *p* < .001, η_*p*_^2^ = .534. This overall memory control effect was a general feature of both studies and did not interact with experiment, *F*(1, 48) = 2.39, *p* = .129, η_*p*_^2^ = .047. Importantly, both lower-trauma *F*(1, 48) = 21.78, *p* < .001, η_*p*_^2^ = .312, and higher-trauma participants, *F*(1, 48) = 33.99, *p* < .001, η_*p*_^2^ = .415, showed robust control effects.

#### Retrieval-induced facilitation

Overall, the facilitated recall arising from repeated retrieval of Think items was statistically comparable across Experiments 1 and 2. The tendency for 16 retrieval attempts to facilitate memories compared with Baseline (0 attempts) was extremely robust, *F*(1, 48) = 102.62, *p* < .001, η_*p*_^2^ = .681, and did not vary by experiment, *F*(1, 48) = 2.77, *p* = .103, η_*p*_^2^ = .055, or with valence of the target, *F* < 1. Facilitation was, however, greater on the SP compared with the IP test, *F*(1, 48) = 62.36, *p* < .001, η_*p*_^2^ = .565, a pattern which did interact with experiment, *F*(1, 48) = 4.86, *p* = .032, η_*p*_^2^ = .092.

Importantly, even in this much larger, integrated sample, facilitation of Think items did not differ reliably across the trauma groups, *F*(1, 48) = 2.21, *p* = .143, η_*p*_^2^ = .044, with both lower-, *F*(1, 48) = 67.49, *p* < .001, η_*p*_^2^ = .584, and higher-trauma, *F*(1, 48) = 37.34, *p* < .001, η_*p*_^2^ = .438, participants experiencing robust facilitation. The comparable facilitation across groups did not interact with experiment, as reflected by a nonsignificant Repetition × Trauma × Experiment interaction, *F* < 1. These findings indicate that our lower- and higher-trauma groups both benefited from the repeated retrieval of memories and that this was consistent across studies.

#### Suppression-induced forgetting

**Figure 3 fig3:**
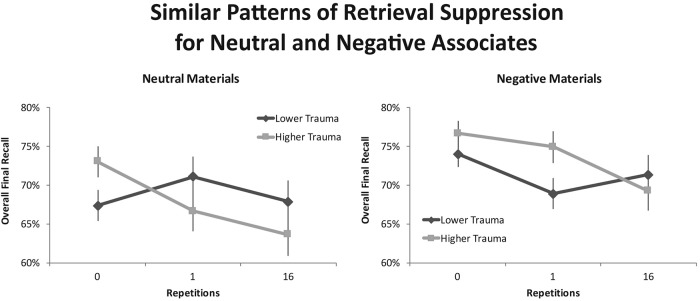
Final No-Think recall results as a function of valence and trauma group. Looking across experiments and test types, similar suppression-induced forgetting in the higher-trauma group was observed across neutral and negative materials. In contrast, the lower-trauma group did not show reliable forgetting in either case. Error bars reflect *SE*s.

The overall suppression-induced forgetting effect was highly robust and comparable across experiments. No-Think items were generally recalled more poorly than were Baseline items, *F*(1, 48) = 12.51, *p* = .001, η_*p*_^2^ = .207, and this effect did not interact reliably with experiment, *F*(1, 48) = 1.02, *p* = .317, η_*p*_^2^ = .021. Critically, as suggested by the individual studies, the apparently greater suppression-induced forgetting for higher-trauma participants was supported by a significant interaction between repetition and trauma history, *F*(1, 48) = *7*.48, *p* = .009, η_*p*_^2^ = .135. Whereas participants with greater experience with trauma displayed significant forgetting of No-Think items across the final tests, *F*(1, 48) = 19.67, *p* < .001, η_*p*_^2^ = .291, the lower-trauma group displayed no reliable evidence of an ability to forget No-Think items, even in this much larger, integrated sample. The trauma-history advantage did not show any evidence of interacting with valence (see Figure 3), *F*(1, 48) = 1.21, *p* = .278, η_*p*_^2^ = .025, or with experiment, *F* < 1. Thus, despite the marginal interaction of trauma and repetition in Experiment 2, the relative difference in suppression-induced forgetting across higher- and lower-trauma groups was statistically indistinguishable across experiments. Indeed, the significant forgetting in the higher-trauma group did not interact with experiment, *F* < 1, nor did the lack of suppression-induced forgetting in the lower-trauma group, *F* < 1.[Fig-anchor fig3]

Thus, despite highly similar overall memory performance and comparable facilitation of Think items, a prior history of more trauma was associated selectively with the enhanced ability to suppress unwanted memories.

#### Comparison of the trauma-history advantage over test type

Although we had hypothesized that the IP test would be more sensitive to differences in inhibitory control and although both studies showed a numerical tendency supporting this, the trauma-history advantage in suppression-induced forgetting still did not vary reliably across our SP and IP tests, *F*(1, 48) = 1.33, *p* = .255, η_*p*_^2^ = .027, despite the increase in sample size. This pattern did not interact with experiment, as reflected in a nonsignificant interaction of Repetition × Trauma × Test type × experiment, *F* < 1. Nevertheless, the lack of an interaction of test type with repetition suggests that the superior forgetting exhibited by participants with more traumatic experience occurs irrespective of test type, and so may have a grounding in improved inhibitory control. Indeed, higher-trauma participants exhibited significant forgetting, regardless of whether No-Think items were analyzed with respect to the SP, *F*(1, 48) = 18.22, *p* < .001, η_*p*_^2^ = .275, or the IP test, *F*(1, 48) = 7.29, *p* = .010, η_*p*_^2^ = .132, consistent with this possibility.

Interestingly, lower-trauma participants exhibited qualitatively different patterns of forgetting on the SP and IP tests. Whereas overall forgetting (collapsed over test type) was not reliable, lower-trauma participants exhibited reliable cue-specific forgetting on the SP test. This cue-specific forgetting pattern in the lower-trauma group was substantiated by a significant interaction of repetition by test type, *F*(1, 48) = 6.04, *p* = .018, η_*p*_^2^ = .112. In contrast to the reliable forgetting seen for this group on the SP test, *F*(1, 48) = 4.70, *p* = .035, η_*p*_^2^ = .089, no impairment was observed on the IP test, *F*(1, 48) = 1.11, *p* = .297, η_*p*_^2^ = .023. This pattern did not interact with experiment, *F*(1, 48) = 1.07, *p* = .306, η_*p*_^2^ = .022. Thus, participants who reported relatively little experience with trauma showed modest forgetting that also appeared to be largely cue-dependent. This suggests that the observed forgetting may not have reflected inhibition (Anderson & Levy, 2009; Anderson et al., 2011; Noreen & de Fockert, 2017; Wang et al., 2015). Taken together, the findings from this integrated analysis suggest that experience with traumatic events may lead to more effective use of inhibitory processes to control unwanted memories, and, as a consequence, to more generalized forgetting of those memories in different cuing situations.

#### Correlational analyses of the integrated sample

We based the foregoing conclusions about trauma history and forgetting on a group analysis approach. To complement this analysis, we also leveraged the additional statistical power afforded with supplementary correlation analyses by treating trauma history as a continuous measure (DeCoster, Iselin, & Gallucci, 2009). These analyses sought to address several issues. First, although our group analyses matched lower- and higher-trauma groups perfectly with regard to our stimulus and test order counterbalancing manipulations, they dichotomized our sample according to the level of reported trauma, discarding information about gradations in trauma that could be helpful in evaluating the generality of the relationship between traumatic experience and inhibition. Second, our group analyses provided numerically suggestive evidence that the IP test is more sensitive to individual differences in inhibitory control capacity that may be related to trauma history. These tendencies were nonetheless not significant. It is possible that the predicted superiority of the IP test in detecting differences in inhibition may surface if our analysis considered more continuous variation in trauma level. Finally, we sought to address potential influences of self-reported depression and Baseline recall level as factors contributing to the putative trauma-history advantage.

To address these objectives, we ran robust correlation tests for our IP and SP measures of suppression-induced forgetting, treating the average trauma score as a continuous measure across our 96 participants. After *z*-transforming the suppression-induced forgetting and trauma scores within each of the stimulus counterbalancing conditions, we used the Robust Correlation Toolbox for Matlab, which down-weights/removes analytically identified bivariate outliers, while statistically correcting for their removal (Pernet, Wilcox, & Rousselet, 2013). Reliability of the Spearman outlier-skipped correlation was determined based on the percentile bootstrap confidence interval (i.e., if the 95% interval includes 0, the null hypothesis of independence was not rejected), a method that is more robust against heteroscedasticity than the traditional *t* test (Pernet et al., 2013). We then subjected the data—stripped of analytically identified bivariate outliers—to an additional partial correlation analysis that examined the relationship between traumatic experience and suppression-induced forgetting while controlling simultaneously for both self-reported depression severity and Baseline recall performance.

**Figure 4 fig4:**
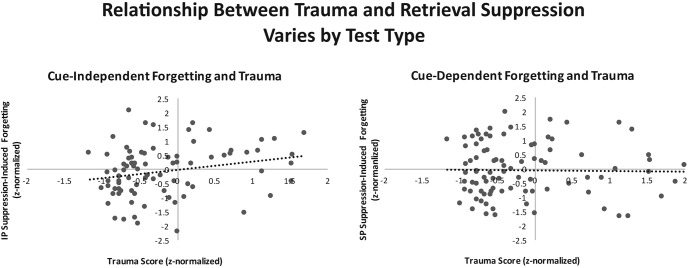
Self-reported trauma from the first 18 years of participants’ lives reliably predicted suppression-induced forgetting on the independent-probe (IP) test of forgetting (left panel). In contrast, the same-probe (SP) measure of suppression-induced forgetting, which is likely contaminated by noninhibitory influences, exhibited no such relationship (right panel). Data were *z*-normalized within counterbalancing conditions. Ten bivariate outliers across the two correlations (automatically identified and statistically corrected for by the Robust Correlation Toolbox; Pernet et al., 2013) were removed from these plots accordingly. Note that positive y-values represent suppression-induced forgetting in these scatterplots, unlike the column graphs found elsewhere in this paper, which represent suppression-induced forgetting with negative values.

In this correlational analysis, we confirmed that increasing levels of reported trauma are associated with more generalized suppression-induced forgetting, as evident by greater forgetting on the IP test. Indeed, as can be seen in Figure 4, the clearest association with trauma history was evident on the IP test (skipped Spearman *r*_s_ = 0.25, bootstrap-based 95% confidence interval [CI] accounting for automatically removed bivariate outliers: [0.03, 0.45]). In contrast, the relationship between trauma history and forgetting on the SP test was not reliable (skipped Spearman *r*_s_ = 0.02, 95% CI [−0.17, 0.23]). Across the nonoutlier data points, the IP and SP correlation coefficients significantly differed according to a one-tailed Meng’s *z* test for correlated correlation coefficients, Meng’s *z* = 1.67, *p* = .048 (Meng, Rosenthal, & Rubin, 1992). This disparity across test types is consistent with prior work indicating that item-specific cuing tests such as the IP test are more sensitive to individual differences in inhibitory control (Anderson & Levy, 2009; Anderson et al., 2011; Schilling et al., 2014; Wang et al., 2015).[Fig-anchor fig4]

Next, we addressed potential influences of self-reported depression and Baseline recall via our supplementary correlational approach. First, it should be noted that in the group analysis, we found that our trauma groups differed in neither BDI scores, *F* < 1, nor Baseline recall, *F*(1, 48) = 3.63, *p* = .063, η_*p*_^2^ = .070, suggesting that these variables are unlikely to explain differences observed in this analysis. Still, we asked whether these factors possibly could account for the observed relationship between trauma history and suppression-induced forgetting; partial correlation analyses (two-tailed tests) suggested not. After controlling for both of these variables, trauma history and IP forgetting remained significantly related, *pr*_*s*_(82) = 0.25, *p* = .025. In contrast, there again was no reliable relationship between trauma history and SP forgetting, *pr*_*s*_(82) = 0.03, *p* = .798.

### Discussion

The integrated analysis helped confirm several important aspects of the case for a trauma-history advantage. First, the combined analysis demonstrated that our participants, who reported experiencing varying amounts of trauma, did not differ reliably in other aspects of performance that were unrelated to inhibitory control, including the ability to benefit from repeated retrieval of Think items—even when considering a much larger sample of participants. The groups did, however, consistently differ in the amount of suppression-induced forgetting they exhibited, a pattern that was statistically equivalent across the two studies. Second, our correlational analyses revealed that the relationship between trauma history and superior forgetting is not tied specifically to our group analysis approach, as it also emerges when one considers continuous variation in the level of trauma. The correlational approach further revealed evidence for the superiority of the IP test in isolating evidence of improved inhibitory control with higher trauma—an effect that was suggestive, though statistically weak, in our group approach. Third, these analyses suggest that differences in Baseline recall or in depression symptoms across trauma groups are unlikely to provide an alternative account of our findings. Finally, the combined analysis yielded evidence that even participants who reported experiencing lower rates of trauma exhibit some forgetting, but that this forgetting is cue-dependent, which may not reflect inhibition.^3^ Taken together, these findings provide strong converging evidence that participants who have lived through more adverse experiences in their lives show improved ability to contend with unwanted memories, as hypothesized.

## General Discussion

Life experience often supplies natural opportunities for refining skills. Whereas few would argue that traumas are good, they may nonetheless drive adaptation that promotes resilience. We claim that controlling one’s memories—remembering what is relevant and setting aside that which is maladaptive—is a valuable skill (see Nørby, 2018, for a review). Here we considered the possibility that trauma experience affords many survivors the opportunity to adapt their memory control abilities, enabling them to better regulate intrusive memories. Though honed in response to particular traumas, these adaptations may generalize to unrelated memories producing lasting benefits that promote long-term resilience in the face of adversity (Seery, 2011). Our data, although correlational in nature, are consistent with this possibility. In our samples, those reporting more traumatic life events prior to age 18 were better at suppressing retrieval of novel associations, regardless of the valence of the suppressed content. We observed evidence of this advantage across two experiments. These individual differences in suppression success are unlikely to reflect biases in reporting to-be-suppressed items or experimenter bias in the administration or scoring of the Think/No-Think task: Offering financial incentives for accurate recall and blinding experimenters/data coders to participants’ trauma history had no effect on the suppression advantage. These findings suggest that those participants who had lived through a greater number of adversities were genuinely better at forgetting experiences they sought not to think about, at least in the context of our controlled laboratory task.

Notably, our higher- and lower-trauma groups performed comparably in other respects, despite differences in retrieval-suppression. The two groups differed neither in Baseline recall nor in the tendency for repeated retrieval to facilitate Think items. Comparable recall after paired-associates training suggests that our trauma groups did not differ markedly in their general motivation to perform our laboratory tasks. Potential motivational differences warrant special scrutiny when the groups under consideration might differ on psychiatric variables, such as depression. However, we found that symptoms of depression were generally low, on average, and were similar across groups—in itself a remarkable finding, given the higher-trauma group’s history of adversity. Importantly, partialing out depression scores did not meaningfully alter the relationship between trauma exposure and suppression-induced forgetting. Taken together, these findings are consistent with trauma experience selectively improving memory inhibition, rather than affecting overall memory performance or motivation. Indeed, the enhanced forgetting in the higher-trauma group was especially evident on a test of cue-independent forgetting designed to isolate the influence of inhibitory control (Levy & Anderson, 2008; Schilling et al., 2014). In contrast to the higher-trauma group, forgetting in the lower-trauma group did not generalize across test types. This cue-dependent pattern observed for the lower-trauma group suggests that the effect reflects mechanisms that promoted retrieval interference at the time of test, more than inhibition of the to-be-suppressed responses.

Interestingly, the current data indicate that the trauma-related memory advantage did not vary according to the valence of the target items being suppressed. Benefits arose for both negative and neutral stimuli. There are reasons to expect that this need not have been the case. For one, people with a greater history of trauma might have shown less benefit of their prior experience for negative material, perhaps because they could have been distracted by relationships of the materials to their own lives. For another, greater benefits for negative content might have arisen if suppressing traumatic intrusions had fostered distinctive component processes not involved in suppressing neutral content. The absence of such an advantage in our data could indicate that the cognitive and neural mechanisms involved in suppressing aversive and neutral content are similar, a possibility compatible with imaging work on retrieval suppression (Gagnepain et al., 2017). Alternatively, differential effects for valenced content might have been observed had we manipulated valence more strongly (e.g., with aversive pictures) or had we used materials tailored to trauma (as is sometimes done in directed forgetting work—McNally, Ristuccia, & Perlman, 2005; Moulds & Bryant, 2002; Noreen & MacLeod, 2013; Zoellner, Sacks, & Foa, 2003). The present pattern of results is, nonetheless, consistent with our theory that trauma history fosters a generalized retrieval suppression skill not limited to affective content.

Although both experiments found superior memory inhibition in participants with a history of adversity, our data may underestimate the true strength of this relationship. Our ability to test this relationship depended on how accurately we could quantify participants’ trauma history. Should individuals with more traumas be better at forgetting, as predicted, this superior memory control ability may actually cause them to underestimate the true frequency of their own adverse experiences. In some cases, this underestimation even might have led them to be misclassified into our lower-trauma group. The impact of this dynamic should depend on the nature of the forgetting induced by retrieval suppression. On the one hand, if suppression primarily reduces involuntary retrievals of unwanted memories or degrades the specificity of episodic details of those events without affecting voluntary access to them (e.g., Noreen & MacLeod, 2013; Stephens, Braid, & Hertel, 2013), suppression may not alter people’s estimates of their own trauma frequency very much. On the other hand, if suppression induces more generalized memory loss for the unwanted events, frequency estimates may be more distorted, reducing differences in forgetting across lower- and higher-trauma groups, increasing the likelihood of potential misclassifications, and reducing correlations between forgetting and trauma history. Although we cannot discern the influence of such an effect in our dataset, this putative bias works against our hypothesis. That we nevertheless observed a robust relationship between trauma history and suppression-induced forgetting is, therefore, all the more remarkable.

### Relation to Existing Work

Two features of the current work make it unique in its capacity to shed light on how cumulative lifetime adversity might affect the ability to control intrusive memories. First, prior studies that examined the relationship of trauma to memory inhibition used variants of the retrieval-induced forgetting or directed forgetting paradigms (Amir, Badour, & Freese, 2009; McNally et al., 2005; Moulds & Bryant, 2002; Zoellner et al., 2003). The relevance of the behavioral circumstances posed by these two procedures to controlling intrusive memories is arguably indirect: Retrieval-induced forgetting concerns the tendency for retrieval of some items to incidentally inhibit other competing memories; directed forgetting concerns the ability to forget an immediately preceding event or set of events, in some cases by terminating encoding of the event. Although both tasks can involve inhibitory control, neither addresses the situation most relevant to combating intrusive memories of trauma: confronting unwelcome reminders and needing to suppress episodic retrieval to prevent awareness of an intrusive memory. We argue that studying retrieval suppression as we do here more directly speaks to the mechanisms relevant to understanding how people regulate intrusions of experiences that are well encoded (Anderson & Hanslmayr, 2014). Second, little work with the directed forgetting procedure has quantified lifetime history of adversity; rather, most such studies have instead focused on diagnostic groups with documented evidence of a disorder (e.g., PTSD or acute stress disorder; e.g., McNally et al., 2005; Moulds & Bryant, 2002; Zoellner et al., 2003). Although findings have been variable, patients with PTSD often show impaired directed forgetting (see, e.g., McNally, 1998), paralleling studies of retrieval suppression in PTSD (Catarino et al., 2015). However, by focusing on a more diverse sample of participants, rather than preselecting on the basis of clinical disorders, we argue that the current study, with its relatively comprehensive accounting of lifetime traumatic experience, is better positioned to characterize the general association between adversity and memory inhibition.

As such, this study adds to a growing body of work indicating that suppression-induced forgetting, as measured by the Think/No-Think paradigm, reflects mechanisms engaged in everyday control over unwanted memories that appear relevant to resilience in the face of unpleasant experiences. For instance, superior self-reported ability to control everyday thoughts not only predicts lower levels of anxiety, depression, and obsessional thinking, but also better suppression-induced forgetting in the laboratory (Küpper, Benoit, Dalgleish, & Anderson, 2014). Other work has revealed negative relationships between suppression-induced forgetting and rumination (Fawcett et al., 2015; Hertel & Gerstle, 2003), trait anxiety (Dieler, Plichta, Dresler, & Fallgatter, 2010; Marzi, Regina, & Righi, 2014), and PTSD symptoms (Catarino et al., 2015). Importantly, individuals who demonstrated superior suppression-induced forgetting in a simple verbal Think/No-Think task were found to experience fewer distressing memory intrusions in the week following exposure to a traumatic film (Streb, Mecklinger, Anderson, Lass-Hennemann, & Michael, 2016). This finding suggests that laboratory measures of retrieval suppression ability are linked to resilience in the face of stressful events. Together with this literature, our current findings suggest that memory control processes engaged during retrieval suppression may play an important role in the adaptation that typically occurs after a trauma and to the building of future resilience. Nevertheless, it would be desirable in future work to directly establish, within the same study, the link between exposure to trauma, superior memory control, and resilience in the face of novel stressors (cf. Seery, Leo, Lupien, Kondrak, & Almonte, 2013).

The current findings cannot distinguish whether traumatic experiences improved participants’ memory control or, conversely, whether good preexisting memory control abilities enabled our higher-trauma participants to overcome their challenging history and make it to college and into our sample. Moreover, without detailed information regarding the severity or developmental timing of the traumas reported by our sample—or whether any led to PTSD or other mental health conditions—we are currently unable to address the extent to which these factors relate to memory suppression abilities and overall resilience. These questions merit careful scrutiny in future work. However, structural adaptations in the right DLPFC in the year following trauma and their relationship to recovery from PTSD (Lyoo et al., 2011) suggest that, at least in adults, retrieval suppression abilities may well undergo adaptation in many individuals, given the right DLPFC’s involvement in this control ability (Anderson & Hanslmayr, 2014). Analogous experience-dependent changes in task-relevant brain structures have been found after practice at golf (Bezzola, Mérillat, Gaser, & Jäncke, 2011), musical performance (Herholz & Zatorre, 2012), left-handed writing and drawing (Wenger et al., 2017), and even working memory (Metzler-Baddeley, Caeyenberghs, Foley, & Jones, 2016) and inhibitory control (Chavan, Mouthon, Draganski, van der Zwaag, & Spierer, 2015)—with these changes predicting behavioral performance (Draganski et al., 2014; May, 2011). To substantiate the hypothesized role of cortical plasticity in improving retrieval suppression, future work should track memory control ability longitudinally in participants who have recently experienced trauma. Doing so could help determine whether behavioral and structural adaptations arising from the trauma predict recovery from intrusion symptoms. If trauma can naturally improve retrieval suppression, as the Lyoo et al. (2011) and current data suggest, it would broaden our understanding of the consequences that trauma has on mental function. Such effects of trauma on memory control are consistent with the broad proposal that psychiatric conditions promote generalized habits of thought (Hertel, 2004).

Given the aforementioned considerations, the current evidence for trauma-related advantages in memory control provides a credible account regarding the cognitive mechanisms that underlie reports of positive effects of adversity on resilience. Seery et al. (2010) noted that, although adversity may have a negative effect on current and future mental health and well-being (e.g., Brewin, Andrews, & Valentine, 2000; Edwards, Holden, Felitti, & Anda, 2003; Golding, 1999), having experienced a moderate level of adversity may be beneficial in developing mechanisms that improve future resilience. In a large scale (*N* > 2000) longitudinal study, they found a nonmonotonic relationship between cumulative lifetime history of adversity and measures of mental health: Moderate adversity was associated with higher self-reported mental health (i.e., lower reported depression/anxiety, lower global distress, lower functional impairment, lower posttraumatic stress) and well-being (i.e., higher rated life satisfaction) compared with having experienced high adversity or no adversity at all. Higher cumulative adversity predicted greater resilience when encountering new traumas years later. Strikingly, the benefits of moderate adversity also arise in laboratory measures of people’s stress responses, including: (a) lower ratings of pain intensity during a cold-pressor task and reduced situational catastrophizing (persistent negative thoughts), and (b) reduced online cardiovascular measures of challenge/threat in response to a stressful intelligence test (Seery et al., 2013). Given evidence supporting a common inhibitory control mechanism mediated by the right prefrontal cortex underlying the suppression of unwanted thoughts, emotions, and actions (Depue, Orr, Smolker, Naaz, & Banich, 2016; Gagnepain et al., 2017; Guo, Schmitz, Mur, Ferreira, & Anderson, 2018; Schmitz, Correia, Ferreira, Prescot, & Anderson, 2017), we suggest that both the data on resilience and the current findings are compatible with the possibility that adversity trains a general inhibitory control mechanism. Although this *inhibition plasticity hypothesis* is plausible, matters are complex, given trauma’s parallel detrimental impacts on well-being. For example, it is unclear why individuals with much higher lifetime adversity show poorer outcomes (Seery et al., 2010) under this account. One possibility is that high, repeated stress could have toxic effects that compromise the neural mechanisms that implement inhibitory control (e.g., GABAergic inhibition in the hippocampus; Schmitz et al., 2017). It remains possible that we, too, would have observed a nonmonotonic relationship between prior adversity and memory suppression had we captured in our sample a wider range of trauma frequency or severity.

### Implications

If the inhibition plasticity hypothesis is correct and real-world conditions precipitate cortical plasticity that supports new habits of thought, this gives hope to potential interventions designed to train memory control, complementing standard cognitive–behavioral therapy. Currently, it is widely believed that avoiding distressing memories exacerbates PTSD symptoms by keeping suppressed memories accessible (e.g., Dalgleish, Hauer, & Kuyken, 2008). Cognitive–behavioral therapy for PTSD is considered effective because it encourages patients to stop avoiding memories and to confront reminders until the traumatic memories become less distressing (Kar, 2011). We suggest an alternative possibility, proposing that there is an important distinction between avoiding reminders, on the one hand, and avoiding the memory (via suppression) after a reminder is confronted, on the other. The former strategy is expected to preserve memories by depriving people of opportunities to forget via inhibitory control, whereas avoidance by successful retrieval suppression is beneficial. Thus, like cognitive–behavioral therapy, retrieval suppression forces people to confront reminders, but has the potential to allow many of these individuals to learn to control awareness of their memories. Given this perspective, training in retrieval suppression may augment the benefits of cognitive–behavioral therapy by enabling patients to confront reminders and redirect to more benign thoughts.

Suggesting that forgetting unwanted memories may be helpful might seem in conflict with the fact that memory lapses for significant aspects of a trauma are considered a hallmark symptom of PTSD, according to the current edition of the *Diagnostic and Statistical Manual of Mental Disorders* (*DSM–5*; American Psychiatric Association, 2013). If so, forgetting is arguably an unhealthy outcome rather than a healthy goal. Two points are worth emphasizing, however. First, as noted previously, research on retrieval-suppression (Catarino et al., 2015), directed forgetting (e.g., McNally, 1998), and even motor response inhibition (Falconer et al., 2008) point to a general deficit in inhibitory control that compromises the effective suppression of intrusive memories in PTSD. If so, then any memory lapses (often referred to as memory fragmentation; see, e.g., Brewin, 2007, 2014) that occur in PTSD may not arise from suppression, but rather from some other process. One possible origin may be compromised episodic encoding of trauma attributable to the impact of extreme stress on hippocampal function in response to the traumatic event (Brewin, Gregory, Lipton, & Burgess, 2010). If stress impairs encoding, then the loss of episodic memory may not itself be a direct cause of PTSD symptoms, but rather an indirect reflection (or marker) of the severity of the original stress response. Second, despite support from a number of studies (see, e.g., Brewin, 2007, 2014, for reviews), the existence of elevated fragmentation/incoherence of trauma memories in PTSD has been challenged (Rubin et al., 2016; but see Brewin, 2016), raising the possibility that memory lapses might not be as reliable a symptom of PTSD as previously thought. Given these observations, we see value in entertaining the possibility that forgetting arising from the successful down-regulation of intrusive memories via retrieval suppression may be a healthy coping response that is dysfunctional in PTSD, leading to difficulty controlling intrusive memories.

Even if retrieval suppression may be healthy by helping to reduce intrusive memories, recent evidence suggests that this benefit also comes with other costs during the adjustment process. As people recover from trauma, they often exhibit generalized memory deficits—including overgeneral autobiographical memory and episodic memory impairments (e.g., Brewin, 2011; Ono, Devilly, & Shum, 2016). Evidence indicates that some forms of retrieval suppression may contribute to these side effects (Dalgleish et al., 2008; Hulbert et al., 2016). For example, when people suppress retrieval, it not only impairs memory for the suppressed trace, but also induces an amnesic shadow over events occurring before and after suppression (Hulbert et al., 2016; Hulbert, Hirschstein, Brontë, & Broughton, 2018). This diffuse memory deficit likely arises from the down-regulation of hippocampal activity caused by retrieval suppression (Anderson et al., 2004) and the resulting disturbance it creates in hippocampal processes like establishing stable, contextualized memories. Such deficits should be greatest in those victims of trauma with intact retrieval suppression ability. Though clearly not desirable, this temporary decline in memory function may represent a trade-off between the need to regulate intrusive memories that disrupt life on one hand, and general mnemonic functioning on the other. Once intrusive memories have been regulated and control processes refined, memory may improve. Indeed, these types of memory deficits following trauma often abate as intrusions decline (Guez et al., 2013).

Exposure to trauma, unfortunately, is pervasive. Data from the World Mental Health Survey Consortium indicate that more than 70% of the nearly 70,000 adults responding from 24 countries across the globe have been exposed to at least one traumatic event in their lifetimes; more than 30% have been exposed to four or more such events (Benjet et al., 2016). Benjet and colleagues went on to report that the overall prevalence of exposure in the United States topped 82% in the years surveyed. Within the U.S. college population from which our participants were drawn, published prevalence estimates range considerably (up to 84%), reflecting variable thresholds in classifying potentially traumatic events (Read, Ouimette, White, Colder, & Farrow, 2011). On the low end, a full 66% of the 3,014 incoming college students assessed across two midsized public universities reported exposure that met a strict criterion based on the DSM (Read et al., 2011). The students reported a mean of 1.5 traumatic events leading up to college matriculation, with a quarter of the overall sample reporting three or more events and 9% meeting criteria for PTSD.

In light of these numbers, it is perhaps not so surprising (albeit quite regrettable) that many of our participants were reportedly exposed to multiple traumas over the first 18 years of their lives. To compare with previous research using the BBTS, we summed the number of items on the traumatic experiences survey for which individuals reported at least one traumatic episode (e.g., Freyd, Klest, & Allard, 2005). This yielded an average of 2.00 (*SD* = 2.16) endorsed items across our entire sample (i.e., irrespective of both experiment and group classification). Broadly, this average is in line with other work using the BBTS. For example, Platt and Freyd (2015) reported that their sample of 124 undergraduates experienced 3.73 total traumas on average before the age of 18—the slightly higher value possibly reflecting that their sample was restricted to females, given the gendered nature of betrayal trauma (DePrince & Freyd, 2002).

It is important to note that we did not have the resources to verify the traumas reported by our participants. As noted previously, however, the incidence rates may, nevertheless, reflect an underestimate of trauma, especially for individuals better able to prevent unwanted memories from surfacing. Some of the reported traumas may reflect the fallout from singular events (e.g., a major hurricane resulting in significant loss), whereas others may reflect repeated episodes of mistreatment (e.g., emotional, physical, or sexual). Our investigation did not seek to assess the relative severity of these episodes. Instead, we aimed to capture an inclusive estimate of the number of events considered by participants to have been traumatic and relate it to suppression-induced forgetting in the laboratory. Indeed, if our hypothesis is correct and painful real-life experiences yield natural opportunities to practice a generalizable skill for controlling memories, the cumulative volume of these adverse experiences may matter more to the relationship than whether the experiences meet the same objective criteria across individuals. Lacking objective and subjective measures of the severity of verified traumatic experiences, we are unable to speak directly to this possibility at present.

### Concluding Remarks

Although approximately 7% to 8% of people develop persistent PTSD in the wake of trauma, most people recover (Kessler et al., 2005). Our findings suggest that traumatic experiences—as horrible as they may be—might naturally contribute to the adaptation of cognitive control skills, thereby improving many survivors’ later resilience, at least those who experienced only moderate levels of trauma. We submit the novel hypothesis that the positive role of suppression in the remission of intrusive memories and its broader benefit to resilience may have been missed in prior work establishing thought suppression as a predictor of PTSD maintenance (e.g., Ehlers et al., 1998). Suppression may indeed increase intrusions and the duration of PTSD in a subsample of trauma survivors with general deficits in inhibitory control (Bomyea & Lang, 2016; Catarino et al., 2015; Falconer et al., 2008), and this may contribute to such an association, together with other maintaining factors. Chronic PTSD sufferers may also be less able to adapt control mechanisms over time, perhaps in part because of genetic variation in cortical plasticity (see Lyoo et al., 2011). But the present work suggests that, for many victims living with trauma, there may be hope in the adage that whatever doesn’t kill you makes you stronger.

### Context of the Research

Clinicians and clinical researchers commonly believe that suppressing unwanted thoughts and memories is a maladaptive coping response to trauma that predicts worse mental health outcomes. We argue that this view overlooks the potential adaptive benefits of suppression in promoting current well-being and in shaping the future capacity for resilience. Whereas it is true that 7% to 8% of people develop persistent PTSD in the wake of trauma, most people recover (Kessler et al., 2005). Given the challenge that recovering from trauma poses, this process seems likely to shape cognitive and emotional processes. Our findings suggest that overcoming such adversity contributes to the adaptation of cognitive control skills, at least in those who experienced only moderate levels of trauma—potentially improving their later resilience. This proposed positive role of suppression in the remission of intrusive memories may have been missed in prior work establishing thought suppression as a predictor of PTSD maintenance (e.g., Ehlers et al., 1998). Suppression may indeed increase intrusions and the duration of PTSD in a subsample of trauma survivors with deficits in inhibitory control (Bomyea & Lang, 2016; Catarino et al., 2015; Falconer et al., 2008), and this may contribute to such an association. Chronic PTSD sufferers may also be less able to adapt control mechanisms over time, perhaps in part because of genetic variation in cortical plasticity (see Lyoo et al., 2011). But for many victims living with trauma, efforts to achieve emotional balance by down-regulating intrusive thoughts may act as a natural form of cognitive training.

## Supplementary Material

10.1037/xge0000461.supp

## Figures and Tables

**Table 1 tbl1:** Final Recall Accuracy

Condition	No-Think repetitions			Think repetitions		
	0	1	16	0	1	16
Experiment 1						
SP test						
Lower trauma	79% [74, 84]	76% [70, 82]	74% [67, 82]	79% [74, 84]	82% [77, 88]	96% [94, 98]
Higher trauma	81% [76, 85]	76% [70, 82]	70% [63, 78]	81% [76, 85]	90% [84, 96]	98% [96, 100]
IP test						
Lower trauma	74% [69, 80]	72% [66, 78]	80% [74, 87]	74% [69, 80]	80% [75, 85]	75% [69, 80]
Higher trauma	78% [72, 83]	74% [68, 80]	73% [66, 80]	78% [72, 83]	81% [76, 87]	75% [69, 80]
Experiment 2						
SP test						
Lower trauma	78% [73, 83]	78% [71, 84]	73% [66, 81]	78% [73, 83]	87% [81, 92]	96% [94, 98]
Higher trauma	82% [77, 87]	76% [70, 82]	73% [65, 80]	82% [77, 87]	92% [86, 97]	97% [95, 91]
IP test						
Lower trauma	51% [46, 57]	54% [48, 61]	51% [44, 57]	51% [46, 57]	61% [56, 67]	60% [54, 66]
Higher trauma	59% [54, 65]	57% [51, 63]	50% [43, 56]	59% [54, 65]	63% [58, 68]	62% [57, 68]
*Note.* Final recall results on the two final test measures (same probe [SP] and independent probe [IP]) as a function of repetitions of No-Think (left) and Think (right) reminders, broken out by trauma history. Values in brackets reflect the 95% confidence interval for the marginal means. Note that the Baseline data (from the 0-repetition condition) are shared across Think and No-Think data.						

## Footnotes

1 The coin toss broke a tie between two participants who reported no trauma. This led, by our group-formation protocol, to one participant with no reported trauma being assigned to the higher-trauma group for the sake of matching counterbalancing and sample size. Excluding these two tied subjects leaves the pattern of suppression-induced forgetting across trauma groups highly similar to what will be reported below. We thus retained the participants to adhere to our specified protocol and include all possible participants.

2 Here we focus our coverage of the results on those main effects and interactions motivated by our *a priori* hypotheses introduced above. The complexity of our overall design, owing to counterbalancing and up to five other factors, makes it impractical to report the results of all possible statistical tests allowed by the design. Cases in which valence or test type significantly qualified our conclusions about suppression-induced forgetting are reported in the main text. It can be assumed that all reported significant repetition effects (or interactions of this factor with trauma or test type) related to suppression-induced forgetting are not moderated by stimulus counterbalancing.

3 An analysis of postexperimental questionnaire questions that probed participants for the strategy that they used for each individual cue word (Levy & Anderson, 2008) revealed highly similar frequencies of reported strategies across the higher- and lower-trauma groups. No differences in strategy frequency were reliable, and strategy reports were not correlated with suppression-induced forgetting. One interpretation of this finding is that, rather than using qualitatively different strategies, the lower-trauma group simply was less able to engage the hypothesized inhibitory control process thought to trigger generalized forgetting.
