# Supplementary material for: What Doesn’t Kill You Makes You Stronger: Psychological Trauma and Its Relationship to Enhanced Memory Control
Source: J Exp Psychol Gen. 2018 Jul 19;147(12):1931–49. doi: 10.1037/xge0000461 (PMC6277128; doi:10.1037/xge0000461)
Supplement: Supplementary file 1 [file zfr999183061so1.zip › zfr999183061so1.docx]

**Supplemental Materials**

**What Doesn't Kill You Makes You Stronger: Psychological Trauma and Its Relationship to Enhanced Memory Control**

**By J. C. Hulbert & M. C. Anderson, 2018, *Journal of Experimental Psychology: General***

[**http://dx.doi.org/**](http://dx.doi.org/10.1037/xap0000174)**10.1037/xge0000461**

| Table S1. | |  |  |  |  |  |  |  |
| --- | --- | --- | --- | --- | --- | --- | --- | --- |
| *Participant Demographics (matched counterbalancing)* | | | | |  |  |  |  |
|  | Experiment 1 | |  | Experiment 2 | |  | Both Experiments | |
|  | Low Trauma | High Trauma |  | Low Trauma | High Trauma |  | Low Trauma | High Trauma |
| Female\|Male | 13\|11 | 13\|11 |  | 15\|9 | 19\|5 |  | 28\|20 | 32\|16 |
| Age (years)* | 20.35 (2.21) | 19.91 (2.47) |  | 19.83 (2.01) | 18.83 (1.09) |  | 20.09 (2.10) | 19.35 (1.93) |
| Trauma Score | 0.08 (0.11) | 0.54 (0.65) |  | 0.02 (0.04) | 0.62 (0.34) |  | 0.05 (0.09) | 0.58 (0.51) |
| # Trauma Events | 0.00 [2.00] | 3.50 [7.00] |  | 0.00 [0.75] | 14.00 [57.25] |  | 0.00 (1.00) | 6.00 (24.75) |
| # Without Trauma | 13 | 1 |  | 18 | 0 |  | 31 | 1 |
| BDI | 6.46 (4.09) | 6.21 (6.11) |  | 4.58 (3.26) | 5.88 (4.16) |  | 5.52 (3.78) | 6.04 (5.17) |

Table S1. Demographics of our final samples across the two experiments, with higher- and lower-trauma groups defined after matching for counterbalancing. The number of female|male participants in each condition are listed. Means (with standard deviations in parentheses) are given for Age*, Trauma Score for events prior to age 18 as assessed on the Brief Betrayal-Trauma Survey’s ([Goldberg & Freyd, 2006](#_ENREF_2)) 0-5 scale, and the Beck Depression Inventory, ([BDI; Beck, Steer, & Carbin, 1988](#_ENREF_1)). Significant differences between low- and high-trauma groups were found based on one-tailed *t*-tests for trauma scores and estimated number of trauma events (but not for BDI). In order to approximate the absolute number of traumatic events experienced before age 18 by our different groups for this table, we additionally converted the six-point trauma scale into a frequency estimate for each of the 12 survey items. We did so by taking the lower bound from the chosen response option’s given range (e.g., a conservative estimate of 21 episodes based on a participant’s rating of “4” on the six-point scale that corresponded to between 21 and 100 episodes of trauma), summing those values across the 12 survey items for each participant, and finally obtaining the median frequency [and interquartile range] across participants within each group. The descriptive results are listed as “# Trauma Events.” We supplemented those results with a count of the number of participants within each group who reported having 0 episodes of the traumatic events surveyed (listed as # Without Trauma). *Three participants in Experiment 1 chose not to divulge their exact age.

| Table S2 | |  |  |  |  |  |  |  |
| --- | --- | --- | --- | --- | --- | --- | --- | --- |
| *Participant Demographics (counterbalancing not matched)* | | | | |  |  |  |  |
|  | Experiment 1 | |  | Experiment 2 | |  | Both Experiments | |
|  | Low Trauma | High Trauma |  | Low Trauma | High Trauma |  | Low Trauma | High Trauma |
| Female\|Male | 14\|10 | 12\|12 |  | 15\|9 | 19\|5 |  | 29\|19 | 31\|17 |
| Age (years)* | 20.21 (2.32) | 20.00 (2.39) |  | 19.54 (1.53) | 19.13 (1.83) |  | 19.88 (1.97) | 19.53 (2.13) |
| Trauma Score | 0.05 (0.06) | 0.57 (0.63) |  | 0.02 (0.04) | 0.62 (0.33) |  | 0.03 (0.05) | 0.60 (0.50) |
| # Trauma Events | 0.00 [1.00] | 4.00 [6.00] |  | 0.00 [0.75] | 14.00 [57.25] |  | 0.00 [1.00] | 6.00 [24.75] |
| # Without Trauma | 14 | 0 |  | 18 | 0 |  | 32 | 0 |
| BDI | 5.67 (4.22) | 7.14 (6.30) |  | 4.46 (3.38) | 6.00 (4.01) |  | 5.06 (3.83) | 6.53 (5.18) |

Table S2. For comparison, demographics of the higher- and lower-trauma groups defined without respect to their counterbalancing condition. As above, the number of female|male participants in each condition are listed. Means (with standard deviations in parentheses) are given for Age*, Trauma Score for events prior to age 18 as assessed on the Brief Betrayal-Trauma Survey’s ([Goldberg & Freyd, 2006](#_ENREF_2)) 0-5 scale, and the Beck Depression Inventory, ([BDI; Beck et al., 1988](#_ENREF_1)). Significant differences between low- and high-trauma groups were found based on one-tailed *t*-tests for trauma scores and estimated number of trauma events (but not for BDI). In order to approximate the absolute number of traumatic events experienced before age 18 by our different groups for this table, we additionally converted the six-point trauma scale into a frequency estimate for each of the 12 survey items. We did so by taking the lower bound from the chosen response option’s given range (e.g., a conservative estimate of 21 episodes based on a participant’s rating of “4” on the six-point scale that corresponded to between 21 and 100 episodes of trauma), summing those values across the 12 survey items for each participant, and finally obtaining the median frequency [and interquartile range] across participants within each group. The descriptive results are listed as “# Trauma Events.” We supplemented those results with a count of the number of participants within each group who reported having 0 episodes of the traumatic events surveyed (listed as # Without Trauma). *Three participants in Experiment 1 chose not to divulge their exact age.

| Table S3 | |  |  |  |  |  |
| --- | --- | --- | --- | --- | --- | --- |
| *Stimulus Set (critical items only)* | | |  |  |  |  |
| Cue Words | |  | Response Words *(and associated independent probes)* | | | |
| Neutral A | Neutral B |  | Neutral | *Neutral IP* | Negative | *Negative IP* |
| clamp | rack |  | shoe | *lace sh_* | agony | *misery ag_* |
| pig | stone |  | lawn | *yard la_* | ugly | *appearance ug_* |
| scalp | gauze |  | boxer | *gloves bo_* | burn | *candle bu_* |
| habit | stitch |  | runner | *track ru_* | cancer | *cure ca_* |
| street | trunk |  | violin | *lessons vi_* | corpse | *anatomy co_* |
| paper | proof |  | theory | *fact th_* | divorce | *lawyer di_* |
| grade | subject |  | grammar | *spelling gr_* | failure | *success fa_* |
| dentist | speech |  | prose | *poetry pr_* | fear | *brave fe_* |
| bathroom | couch |  | journal | *diary jo_* | filth | *grime fi_* |
| umbrella | flag |  | football | *tackle fo_* | funeral | *ceremony fu_* |
| hair | soup |  | custom | *ritual cu_* | garbage | *bag ga_* |
| arm | line |  | tennis | *court te_* | addict | *crack ad_* |
| spray | moist |  | brandy | *flask br_* | infection | *ear in_* |
| hole | space |  | echo | *canyon ec* | jail | *cell ja_* |
| hammer | sight |  | noise | *crackle no_* | jealousy | *envy je_* |
| history | news |  | era | *century er_* | lie | *rumor li_* |
| ketchup | cleaner |  | vest | *sweater ve_* | maggot | *insect ma_* |
| crib | cat |  | gender | *lesbian ge_* | neglect | *ignore ne_* |
| shadow | fall |  | leaf | *plant le_* | nightmare | *dream ni_* |
| rag | can |  | pitcher | *water pi_* | poverty | *welfare po_* |
| privacy | episode |  | clarinet | *woodwind cl_* | quarrel | *dispute qu_* |
| twine | phone |  | bell | *chime be_* | rape | *date ra_* |
| butter | fish |  | wine | *cheese wi_* | rotten | *spoil ro_* |
| mark | order |  | designer | *fashion de_* | slap | *hit sl_* |
| swamp | slice |  | mushroom | *fungus mu_* | suicide | *ledge su_* |
| bus | tank |  | bang | *crash ba_* | hostage | *captive ho_* |
| detail | plan |  | method | *scientific me_* | traitor | *spy tr_* |
| apple | idol |  | art | *craft ar_* | sin | *original si_* |
| rim | pace |  | drum | *snare dr_* | ulcer | *stress ul_* |
| egg | morning |  | phase | *transition ph_* | vomit | *gag vo_* |

Table S3. Critical word pairs used in both experiments, as well as the associated independent probes. In Experiment 2, the word stems associated with the independent probes (IPs) were scaled back to a single letter. Cue words (which were always neutral in valence) from sets A and B were assigned to their negative and neutral complements, in a counterbalanced fashion across participants. Six additional pairs (not presented above) were employed as fillers.
